# Long-Term Clinical Outcomes of 3D-Printed Subperiosteal Titanium Implants: A 6-Year Follow-Up

**DOI:** 10.3390/jpm14050541

**Published:** 2024-05-18

**Authors:** Neculai Onică, Dana Gabriela Budală, Elena-Raluca Baciu, Cezara Andreea Onică, Gabriela Luminița Gelețu, Alice Murariu, Mihail Balan, Mihaela Pertea, Carmen Stelea

**Affiliations:** 1 Department of Surgery, Faculty of Dental Medicine, University of Medicine and Pharmacy “Grigore T. Popa”, 700115 Iasi, Romania; onica_neculai@d.umfiasi.ro (N.O.); gabriela.geletu@umfiasi.ro (G.L.G.); alice.murariu@umfiasi.ro (A.M.); mihail.balan@umfiasi.ro (M.B.); carmen.stelea@umfiasi.ro (C.S.); 2 Department of Implantology, Removable Dentures, Dental Technology, Faculty of Dental Medicine, University of Medicine and Pharmacy “Grigore T. Popa”, 700115 Iasi, Romania; dana-gabriela.bosinceanu@umfiasi.ro; 3 Department of Plastic Surgery, Faculty of Medicine, University of Medicine and Pharmacy “Grigore T. Popa”, 700115 Iasi, Romania; mihaela.pertea@umfiasi.ro

**Keywords:** subperiosteal implants, edentulous, complications

## Abstract

As an alternative to regenerative therapies, numerous authors have recently proposed bringing back subperiosteal implants. The aim of the study was to present our clinical experience with a subperiosteal jaw implant that needs minimal bone preparation and enables the rapid implantation of prosthetic teeth in edentulous, atrophic alveolar bone. The research included 36 complete or partial edentulous patients (61 subperiostal implants) over a period of 6 years. To create the patient-specific subperiostal implants design, DentalCAD 3.0 Galway software (exocad GmbH, Darmstadt, Germany) was used and fabricated with a Mysint 100 (Sisma S.p.A., Piovene Rocchette, Italy) by titanium alloy powder. The results showed that only 9 of the 36 cases were successful at 6-year follow-up, while 27 cases had complications, including exposure of the metal frame (early or delayed), mobility of the device prior to the first 4–6 months, and late mobility due to recurrent infections and progressive structure exposure; 1 case failed for reasons unrelated to the device. This study indicated that the prudent application of fully customized subperiosteal jaw implants is a dependable alternative for the dental rehabilitation of atrophic edentulous cases that necessitate bone grafts for traditional fixed dental implant solutions.

## 1. Introduction

Extremely edentulous maxilla and mandibular atrophy have made dental prosthesis rehabilitation very difficult [1]. Due to the high prevalence of compromised elderly patients with serious medical conditions, many patients with atrophic edentulous jaws, particularly the most severe Cawood and Howell Class VI cases [2], may never have access to a fixed dental prosthetic solution that would greatly enhance their quality of life. The long-term survival rate of endosseous implants is considered to be remarkable [3,4]. However, a certain quantity and quality of bone are needed for its installation [5]. Bone grafts, jaw osteotomies, sinus lifts, distractions of the alveolar ridge, zygomatic implants, and barrier membranes are traditionally used in complex reconstructive surgery for dental rehabilitation of atrophic edentulous jaws and alveoli, which is necessary for patients undergoing oncology or post-traumatic treatment [2,6,7]. The main drawbacks, without a doubt, are the long times between treatment initiation and final prosthesis delivery, the high morbidity rate, and the complex and time-consuming procedure [8,9,10]. 

Oral and maxillofacial surgery has made significant strides forward with the integration of computer-aided design (CAD) and computer-aided manufacturing (CAM) systems. This technological leap has not only optimized the fabrication process but has also been a catalyst for innovation within the field. Among the numerous advances, the development of patient-specific implants (PSIs) stands out. These implants are tailored to the unique anatomical requirements of each patient, providing superior fit and function compared to traditional implants. 

The advent of a new generation of subperiosteal patient-specific implants is one of the most noteworthy outcomes of this technological hype [11]. These implants offer a viable solution for patients with inadequate bone height who are not candidates for traditional endosseous implants without undergoing extensive bone grafting or augmentation procedures. However, the challenges related to inadequate mucoperiosteal integration are largely determined by the gingival biotype, which affects the quality and the volume, pertaining to the quantity of the overlying soft tissues [12]. Typically, significant bone resorption is accompanied by comparable reductions in soft tissue, supporting the notion that the soft tissue follows the bone. Additionally, given that subperiosteal implants are prescribed for cases with severe bone loss, the quality of the underlying bone is crucial for the long-term viability of the implants.

The surge in popularity of these subperiosteal PSIs can be attributed to their minimally invasive nature and the reduced surgical morbidity associated with their placement. The use of CAD/CAM technology in their design ensures highly accurate adaptation to the bone surface, which is critical for the stability and long-term success of the implant. Furthermore, this technology has facilitated the manufacturing of these complex structures with precision and efficiency, allowing for shorter production times and lower costs. Worldwide studies [13,14,15,16,17,18] have reported promising outcomes, with numerous patients experiencing enhancements in oral function, aesthetic satisfaction, and overall quality of life. 

Implant failure can be attributed to suboptimal adaptation at the time of surgical placement, leading to potential implant mobilization or instability, structural fracture, infectious processes, or a diminution of osseous support in the absence of infection. 

The objective of the research was to report the clinical findings obtained from a six-year follow-up of clinical cases, focusing on the complications of 3D-printed subperiosteal titanium implants used in the fixed prosthetic restoration of atrophic jaws.

## 2. Materials and Methods

### 2.1. Study Design and Patient Selection Criteria

During the interval from March 2017 to January 2018, 54 patients presented to the private Oral and Maxillofacial Surgery Centre with total and partial edentulism. However, 36 patients were eligible for treatment with custom fabricated DMLS (Direct Metal Laser Sintering) titanium subperiosteal implants.

Eligibility for participation was based on the following inclusion criteria:

- Age over 60 years, or younger individuals with severe bone loss, thin zygomas (<4 mm), or reduced vertical height, making it extremely challenging to place two zygomatic implants on the same side;
- Stable general and oral health status;
- Good oral hygiene;
- Complete or significantly partial edentulism accompanied by severe bone atrophy, which precludes the placement of standard-size implants;
- Opting out of bone regeneration procedures;
- Consent to attend postoperative follow-up appointments.

The exclusion criteria were as follows:

- Age under 60 years, except for the selected younger patients;
- Diagnosed with systemic diseases or receiving pharmacotherapy that contraindicates surgical intervention, including the following:
  - Immunocompromised state;
  - Uncontrolled diabetes mellitus;
  - Neoplasms of the head and neck region;
  - Undergoing bisphosphonate therapy;
  - Inadequate oral hygiene.
- Lifestyle habits such as tobacco use or bruxism;
- Less severe cases of partial or complete edentulism where the placement of standard-sized dental implants is feasible;
- Incapacity or unwillingness to adhere to requisite postoperative follow-up protocols.

The primary causes of tooth loss and jaw atrophy were severe periodontal disease and failure of conventional implants, which involved ongoing bone resorption around older implants.

All patients gave their informed consent after discussing the diagnosis, the outcomes with and without intervention, detailed therapeutic options, and the advantages, inherent risks, and possible complications associated with the treatment.

### 2.2. Pre-Surgical Cone-Beam Computed Tomography (CBCT)

**Figure 1:**
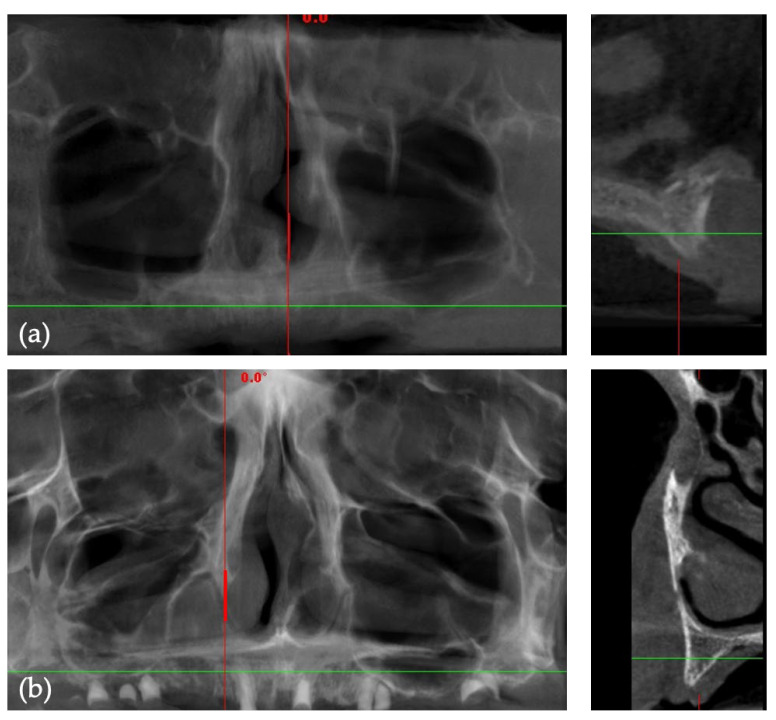
Pre-surgical CBCT images: (**a**,**b**) panoramic (green lines) and cross-sectional (red lines) views.

Prior to clinical intervention, each participant underwent cone-beam computed tomography (CBCT) imaging. These CBCT scans were obtained utilizing Green X (Vatech, Hwaseong-si, Korea), operating under the parameters of a tube voltage of 60–99 kVp, tube current of 4–16 mA, and a focal spot size of 0.5 mm (Figure 1).

### 2.3. Design and Production of Patient-Specific Subperiostal Implants

Utilizing Exoplan 3.0 Galway software (Exocad GmbH, Darmstadt, Germany), the collected Digital Imaging and Communications in Medicine format (DICOM) data from the CBCT scans were processed to reconstruct the residual anatomical structure of the patient’s bone in three dimensions; we subsequently saved the model as a standard tessellation (STL) file. Appropriate threshold values were meticulously selected to accurately render the cortical boundaries of the remaining bone. This process also included the strategic placement of the osseous fixation screws. Subsequently, the STL file underwent refinement within Exocad Galway 3.0 software (Exocad GmbH, Darmstadt, Germany), where descattering, removal of irregularities, and rectification of mesh anomalies were performed, thereby enhancing the visualization of the requisite prosthetic emergence profile and facilitating superior implant design.

**Figure 2:**
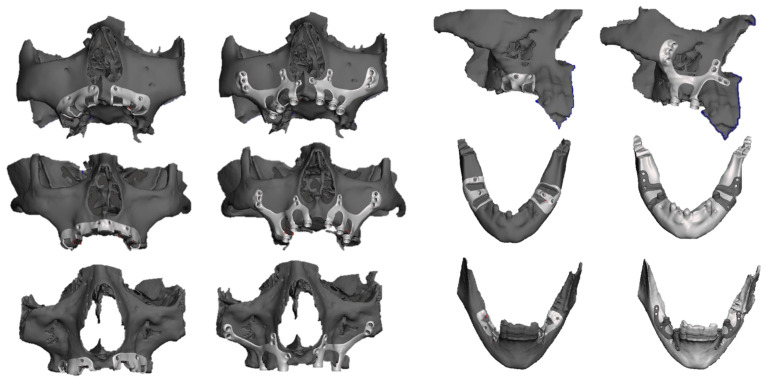
Three-dimensional virtual planning of different subperiosteal structures.

Continuing within the same digital framework, the surgical cutting guide and the implant framework were constructed based on the STL files. Precise locations for the osteosynthesis screws were designated, and internal threading was incorporated to accommodate the multi-unit abutments. The edges were refined, surfaces were smoothed, angles were rounded, and the congruency of the implant with the bone surface was verified (Figure 2).

The comprehensive final designs were prepared for the manufacturing phase and subsequently sent to be printed with a DMLS system (Mysint 100, Sisma S.p.A., Piovene Rocchette, Italy) and titanium alloy powder (PowderRange Ti64, Carpenter Technology Corporation, Philadelphia, PA, USA). In total, 61 hybrid prostheses were fabricated (Table 1), and before packing and delivering, acid etching, plasma cleaning, and autoclave sterilization were performed.

### 2.4. Surgical Procedure and Prosthetic Treatment

**Figure 3:**
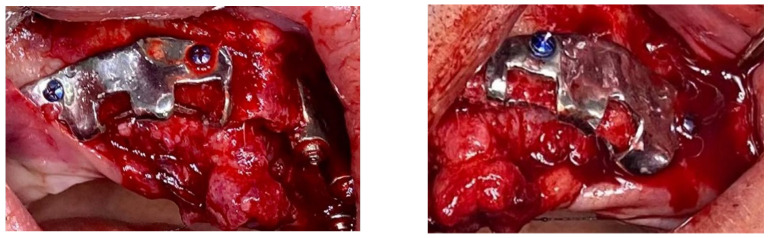
Intraoperative view of bone reduction guide.

The surgical procedures were typically conducted under local anesthesia (4% articaine with 1:100,000 epinephrine, Ubistein, 3M ESPE, St. Paul, MN, USA), with chairside monitoring and sedation administered by an anesthesiologist. The operative technique involved a crestal incision followed by adequate reflection of the periosteal flap, facilitating the placement of the implant. The bone-cutting guide was utilized for precise adaptation and positioning of the structure (Figure 3). Subsequent bone reduction and implant insertion were performed upon removal of the guide.

**Figure 4:**
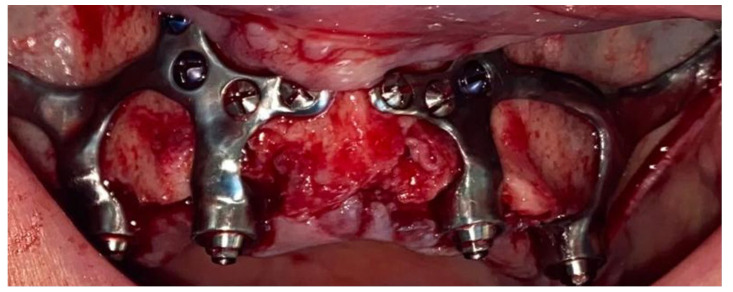
Intraoperative image showing two subperiosteal implants positioned and stabilized with screws.

When affixing the structure to the bone, self-drilling screws (Medicon eG, Tuttlingen, Germany) with a diameter of 2 mm were typically employed (Figure 4).

The screws’ lengths varied, extending from 5.5 to 9 mm in the paranasal and sub-spinal regions and from 11 mm to 13 mm within the zygomatic bone. Despite the self-drilling nature of the screws utilized in zygomatic applications, pre-drilling was executed to prepare the site. The protocol for screw insertion generally commenced with one screw at the anterior pillar, followed by the placement of 2 to 3 screws into the zygomatic bone, and concluding with the remaining screws in the paranasal and sub-spinal areas.

Occasionally, contingent upon clinical requirements, 1 to 2 palatal screws were positioned on each side. In the mandibular procedures, placement of screws on the lingual side was not practiced. Following the structural placement, a slow-resorbing membrane (Mucoderm) was applied to augment soft tissue thickness and prevent or delay potential exposure. The surgical site was closed with resorbable sutures. 

For the first week after surgery, oral antibiotics (Augmentin, Glaxo Wellcome, Mayenne, France), analgesics, anti-inflammatories, and 0.12% chlorhexidine mouthwashes were administered two or three times a day.

The prosthetic rehabilitation involved taking impressions within 2 to 7 days after the surgical procedure for the provisional acrylic screw-retained prostheses. The impressions were obtained either traditional, using open-tray and polyether impression material (Impregum, 3 M ESPE, St. Paul, MN, USA) or digitally, with the CEREC Primescan intraoral optical scanner (Dentsply Sirona, Hanau, Germany). After 6 to 12 months following surgery, the final fixed restorations were performed using CAD/CAM milling technology. 

Postoperative CBCT imaging was performed to ensure accurate positioning of the structure and to verify the precise placement of the screws.

### 2.5. Evaluating Complications and Implant Survival

In this study, the evaluated metrics encompassed immediate, early, and late postoperative complications. The last two types were further classified into minor and major complications. Minor complications included issues like exposure and infection. Major complications encompassed more severe problems such as recurrent infection, mobilization, and fracture of the structure, leading to the structure removal.

Immediate complications were defined as any immediate or secondary adverse event, such as discomfort, swelling, edema, or hemorrhage, that manifested within the initial two weeks after surgery, prior to the placement of the initial temporary restoration. These complications were of a biological origin.

Early complications refer to any issues that occurred during the period between the provisional and final restoration of a dental procedure. The three main complications may include infection, exposure, and mobilization. Common contributing factors to these problems often include improper fitting dental prostheses, an insufficient number of retention units, compromised bone density, and inadequate oral hygiene practices.

Late complications were any biological issues that developed following the delivery of the final prosthetic restoration until the 3-year follow-up. These issues could be of biological origin. Serious and/or recurring infections, with exudation or suppuration, discomfort, swelling, or pus development, with or without radiographic evidence of bone loss, are examples of late biological consequences. Late mobilization of the structure is due to the screw loosening after recurrent infections and insufficient mucoperiosteal and bony integration.

Subperiosteal implants deemed successful at the 6-year follow-up exhibited uninterrupted function without biological complications such as mucositis, exposure, or recurrent infection. Conversely, implants that required removal were categorized as failures. The survival rate included successful implants as well as those with manifestations of exposure and one to two infectious episodes, which remain under clinical monitoring.

### 2.6. Statistical Analysis

Statistical analysis was performed using IBM Statistical Package for the Social Sciences (SPSS) software (SPSS Inc., Chicago, IL, USA, version 26.0 for Windows). Descriptive data were analyzed using frequency and crosstabulation.

## 3. Results

The gender distribution in the study sample was 17 females (47.2%) and 19 males (52.8%). The ages ranged from 38 to 71 years, with a mean age of 61.9 ± 11.7 years. The characteristics of the study participants are presented in Table 2.

**Figure 5:**
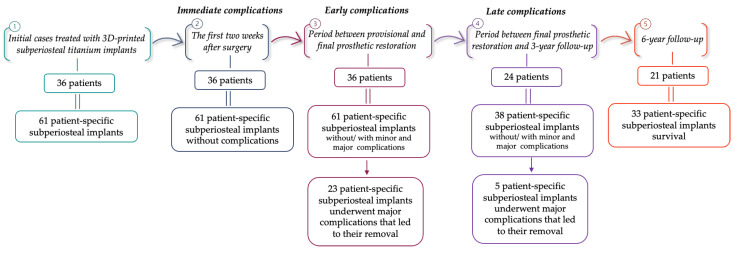
Schematic representation of the evolution of the cases included in the study.

From the initial 36 patients, salvage of the PSIs was not an option in 15 cases due to severe infectious episodes, pain and discomfort, and progressive mobility (see Figure 5 and Table 3). Some of the cases with more stable results but with progressive exposure were addressed between 18 and 24 months with very poor outcomes. The surgical interventions only hastened the progression of the exposure, leading to further complications and making salvage surgery basically useless. 

**Figure 6:**
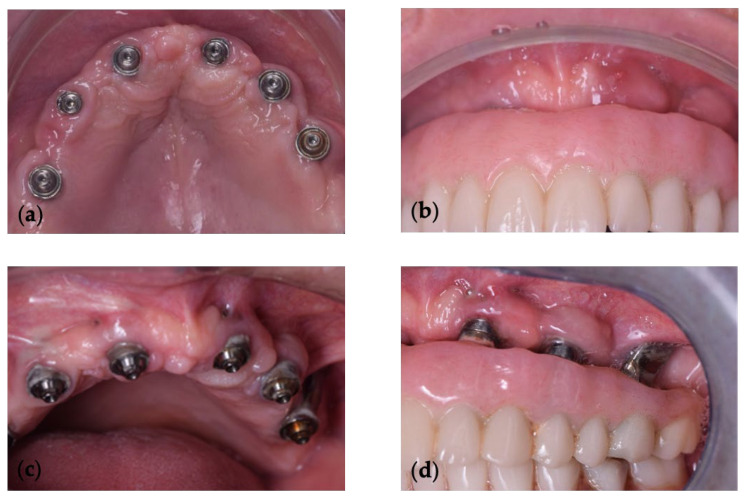
Intraoral aspect at (**a,b**) 24 months and (**c,d**) 42 months.

After a 24-month period, progressive exposure was observed in eight patients in areas where adequate oral hygiene could not be maintained (Figure 6). 

**Figure 7:**
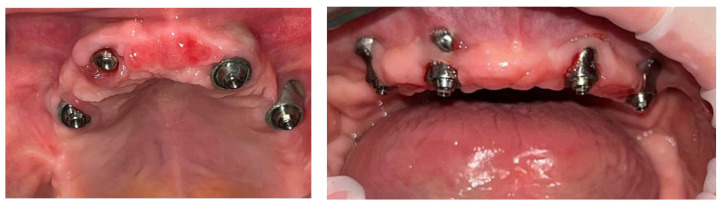
Intraoral examination at 6-year follow-up: minor complication involving soft tissue recession and exposure of structural supports.

At the 6-year follow-up, structural removal was necessary for 15 patients. Currently, 12 patients remain under surveillance, while 9 patients have shown no complications. In the group under observation, we noted a soft tissue recession ranging from 2 to 4 mm, leading to slight exposure of the structural supports, predominantly on the buccal side (Figure 7). 

The incidence of early complications (both minor and major) was 67.2% for a total of 61 PSIs, while the incidence of late complications was 68.4% for 38 PSIs. Statistical analysis revealed no significant differences in the occurrence of complications based on patient gender and age (Table 4). However, there were statistical differences in late complications and 6-year follow-up, associated with the implant location.

## 4. Discussion

The purpose of this publication was to convey our 6-year clinical observations with a novel, digitally custom-designed subperiosteal jaw implant. This technique was mainly addressed to patients with severe bone resorption in which bone grafting would be highly unpredictable due to the decreased osteogenic capacity of the recipient site, especially those with Cawood and Howell Class IV to VI edentulous ridges [2,7,18,19].

Historically, lost wax casting was the method of choice for manufacturing subperiosteal implants. However, issues with this process could compromise the clinical outcome and the final implant-to-bone fit [20]. In recent years, the adoption of CAD-CAM technologies and advanced manufacturing techniques like Direct Metal Laser Sintering (DMLS) and Selective Laser Melting (SLM) have facilitated the design and fabrication of these implants. These additive manufacturing processes allow for high precision in replicating the digitally generated STL file, significantly enhancing the fit and reducing human error [21,22,23,24]. Modern tools like DICOM cone-beam CT images and software such as Exoplan and Exocad 3.0 Galway have revolutionized the design process in virtual space, eliminating the need for physical alterations [25,26].

The clinical protocol has evolved from taking direct impressions of surgically exposed bone to installing heavy frames designed through digital workflows. This change has not only improved the healing process but has also enhanced the success rates of these devices [23,27]. The literature suggests that when standard implants are not suitable or extensive bone regeneration procedures are necessary, customized subperiosteal implants should be considered [27,28]. These implants are particularly beneficial as they can be loaded immediately, accelerating the recovery of function and quality of life for patients [29,30,31]. Typically, these implants support either fixed full-arch prostheses or partial-arch restorations and have successfully rehabilitated edentulous patients when used in retained overdentures [10].

Furthermore, multiple centers have documented innovative designs and applications of the new generation subperiosteal implant, showing the potential of multiple separate units to reconstruct a full arch, whereas a single unit may suffice for partial edentulous arches [13,14,15,16,17]. Our device is composed of 2 units to reconstruct a full arch and a single unit to reconstruct a partial edentulous arch. However, despite positive reviews, the literature lacks comprehensive data, with many studies reporting on a limited number of patients followed for insufficient periods [13,15]. The absence of multicenter clinical trials with long-term outcome data raises questions about the safety and efficacy of these implants on a broader scale.

A complete preoperative diagnosis remains crucial for a successful outcome. Prior to surgical treatment, thorough patient history collection, intraoral and extraoral examinations, high-quality CT scans, and meticulous prosthetic planning are essential to mitigate risks of biological and mechanical complications such as soft tissue dehiscence, peri-implantitis, or fractures. The integration of printed models and digital planning also plays a significant role in minimizing fitting issues, which are critical for the structural stability of the implants [28]. It is also essential to consider both surgical and prosthodontic factors meticulously, ensuring that the final prosthesis is designed with precision. Although some studies have suggested that cone-beam computed tomography provides adequate results [28], a high-quality CT scan is generally required for these fully customized implants to ensure they are perfectly tailored to the patient’s anatomy [32].

Our study found a 62.3% survival rate for implants (including minor complications) in the first 3 years, with a 45.9% failure rate at the 6-year follow-up. This contrasts with findings from other studies. In a pilot study involving 16 patients, Nemtoi et al. [33] observed that after 6 months, only one custom-made DMLS subperiosteal maxillary implant failed, while 75% of the implants demonstrated a good to excellent fit. Mangano et al. [34] reported a perfect survival rate in their one-year study of ten patients, with a 10% incidence of early complications and a 20% incidence of late complications. Similarly, a prospective clinical investigation by Mounir et al. [35] reported a 100% survival rate after one year among ten patients, divided into two groups based on the material of the subperiosteal implants: Ti6Al4V alloy and Polyetheretherketone (PEEK), with five patients in each group. Furthermore, the retrospective analysis by Cerea et al. [21], which included the largest cohort of 70 patients and had the longest follow-up period of at least two years, showed a 95.8% implant survival rate with a 1.4% incidence of biological problems and an 8.9% incidence of mechanical issues.

Regarding gender, we found no difference in outcomes between male patients and females, even though all the females were postmenopausal (without bisphosphonate therapy). The explanation might be that, although females experience accelerated alveolar bone resorption and decreased bone mineral density due to osteoporosis, the impact may be offset by poorer hygiene practices and less frequent maintenance visits observed in male patients. 

In younger individuals (under 60 years old), we observed inflammatory phenomena with an early onset and increased aggressiveness compared to others. This aspect can be attributed to an enhanced reactivity of the body to foreign bodies.

**Figure 8:**
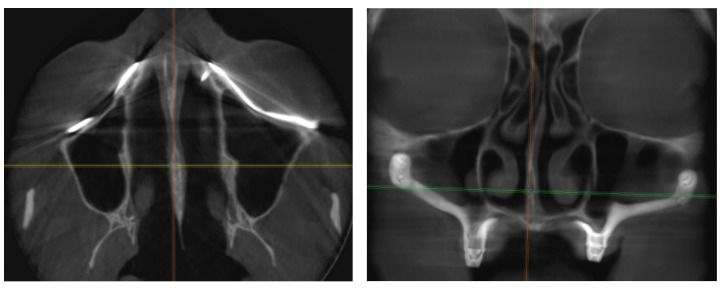
Radiographic images of a properly fitted dental implant at the two-year follow-up.

**Figure 9:**
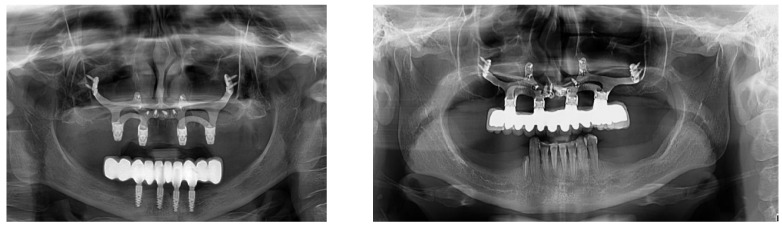
A sufficient number of screws can prevent future mobilization of the structure.

Based on our findings, early complications may arise from several directly involved factors, some of which are related to the structure of the implant, while others pertain to the patient. In terms of the structural integrity, there are two critical aspects that ensure the primary and long-term stability of the subperiosteal implant:

1. The precise fitting of the titanium frame to the bony surface (Figure 8). CAD-CAM technology typically provides accurate structures if the equipment, from CBCT to additive manufacturing, is calibrated correctly. Thus, the human factor remains the primary variable affecting the outcome.

2. A sufficient number of screws should be placed in the most optimal positions within thick pillars and buttresses (Figure 9). The screws may be as long as the clinical situation permits, with a preference for bicortical placement. We recommend using at least four screws in the anterior pillar, with at least two positioned at the lower pole of the piriform rim, measuring 2/9 mm. Additionally, at least two screws should be anchored in the zygomatic bone, with a minimum size of 2/11 mm. Stefano et al. [36] suggested in their case report that for the canine fossa and zygomatic bone, screws measuring 6 to 8 mm are necessary to allow for stable fixation. Self-drilling screws appear to provide better primary stability than self-tapping screws. Regardless of the screw type, predrilling at the zygomatic bone anchorage is essential.

3. The number of struts can significantly impact long-term soft tissue stability. Faster and more pronounced exposure has been observed in structures with three struts on a hemiarch compared to those with two struts.

The present study is limited by the small number of patients included and the absence of consensus on the design of PSIs, their indications based on bone quality, and the optimal positioning and number of screws required to achieve both primary and long-term stability. Despite promising advancements in subperiosteal implant technology have shown promising results, the limited clinical data available highlight the need for further research. Future studies should focus on increasing the sample size and extending follow-up periods to fully assess the viability of these advanced implant solutions.

## 5. Conclusions

Our results, showing a 25% (9 patients from 36 initial) success rate at the 6-year follow-up, indicate that subperiosteal implant-supported hybrid prostheses, designed via digital planning and guided surgery, have proven unsatisfactory and remain a questionable option for long-term treatment. There is a need for additional research into the biocompatibility of the materials used, the design of the structure, and how the body reacts to it. 

## Figures and Tables

**Table 1:** Characteristics of PSIs.

Location	Type	Number of Subperiosteal Structures	Number of Struts
Maxillary	Full arch	26	3 struts on a hemiarch
		20	2 struts on a hemiarch
	Partial Uniterminal arch	2	2 struts
Mandibular	Partial Biterminal arch	2 (×2)	2 struts on a hemiarch
	Partial Uniterminal arch	9	2 struts

**Table 2:** Characteristics of initial study participants (N = 36).

Variables	Number	Percent [%]
Gender		
Male	19	52.8
Female	17	47.2
Age (mean ± SD ^1^)	61.9 ±11.7	
60 years and under	5	13.9
Over 60 years	31	86.1
Edentulism		
Completely or partially edentulous maxilla	25	16.7
Partially edentulous mandible	11	13.9
Patient-specific implant location		
Maxillary	48	78.7
Mandibular	13	21.3

^1^ SD: standard deviation.

**Table 3:** Characteristics of PSI complication.

Variables	Number	Percent [%]
Immediate complications		
Without complications	61	100
Implant survival	61	100
Early complications		
Without complications	20	32.8
Minor complications	18	29.5
Major complications with structure removal	23	37.7
Implant survival	38	62.3
Late complications		
Without complications	12	31.6
Minor complications	21	55.3
Major complications with structure removal	5	13.2
Implant survival	33	54.1
6-year follow-up		
Patient-specific implant success	12	36.4
Monitoring the patient-specific implant	21	63.6
Implant survival	33	54.1

**Table 4:** Aspects of the complication’s appearance.

Variables		Early Complications (N, %)			Late Complications (N, %)			6-Year Follow-up (N, %)		
		Without	With	*p*-Value	Without	With	*p*-Value	Without	With	*p*-Value
Gender	Male	7 (36.8%)	12 (63.2%)	0.637	4 (30.8%)	9 (69.2%)	0.459	4 (36.4%)	7 (63.6%)	0.528
	Female	5 (29.4%)	12 (70.6%)		5 (45.5%)	6 (54.5%)		5 (50%)	5 (50%)	
	Total	12 (33.3%)	24 (66.7%)	-	9 (37.5%)	15 (62.5%)	-	9 (42.9%)	12 (57.1%)	
Age	≤60 years	2 (40%)	3 (60%)	0.733	1 (50%)	1 (50%)	0.803	1 (50%)	1 (50%)	0.830
	>60 years	10 (32.3%)	21 (67.7%)		9 (40.9%)	13 (59.1%)		8 (42.1%)	11 (57.9%)	
	Total	12 (33.3%)	24 (66.7%)	-	10 (41.7%)	14 (58.3%)		9 (42.9%)	12 (57.1%)	
Location	Maxillary	16 (33.3%)	32 (66.7%)	0.861	4 (15.4%)	22 (84.6%)	0.002 *	4 (18.2%)	18 (81.8%)	0.002 *
	Mandibular	4 (30.8%)	9 (69.2%)		8 (66.7%)	4 (33.3%)		8 (72.7%)	3 (27.3%)	
	Total	20 (32.8%)	41 (67.2%)	-	12 (31.6%)	26 (68.4%)		12 (36.4%)	21 (63.6%)	-

* Significance level of 0.05 (Chi-square test).

## Data Availability

The original contributions presented in the study are included in the article, further inquiries can be directed to the corresponding authors.
